# Modulating Magnesium Ion Release for Dual Enhancement of Gel Properties and Nutrient Retention in Selenium-Enriched Tofu

**DOI:** 10.3390/foods15030452

**Published:** 2026-01-27

**Authors:** Fute Du, Tingting Tang, Jinxiaohan Zhang, Xiaoke Yan, Ying Xin, Yujie Su, Ming Zhang, Yuanqi Lv

**Affiliations:** 1 College of Food Science and Technology, Henan University of Technology, Zhengzhou 450001, China; 2 Chongqing Key Laboratory of Economic Plant Biotechnology, College of Landscape Architecture and Life Sciences, Chongqing University of Arts and Sciences, Yongchuan, Chongqing 402160, China; 3 School of Food Science and Technology, Jiangnan University, Wuxi 214122, China; 4 Food Research Center, Zhongyuan Institute, Zhejiang University, Zhengzhou 450001, China

**Keywords:** selenium-enriched tofu, emulsion coagulant, shear rate, magnesium ion release, gel properties, selenium retention, nutrient retention

## Abstract

Traditional rapid coagulation processes often compromise the quality of selenium-enriched tofu, leading to suboptimal texture and substantial nutrient loss. This study regulated the gel properties and nutrient retention of selenium-enriched tofu by controlling magnesium ion (Mg^2+^) release from a water-in-oil (W/O) emulsion coagulant through shear rate adjustment (6000–12,000 r/min). The results demonstrated that at the optimal shear rate of 8000 r/min, sustained Mg^2+^ release facilitated the formation of a homogeneous and dense microstructure accompanied by significantly increased β-sheet content. Compared with conventional methods, the resulting tofu exhibited significant improvements in resilience (increased from 38.73% to 42.54%), water-holding capacity, and nutrient retention, with the selenium content rising from 44.42% to 54.57%. Conversely, deviations from this optimal condition produced either mechanically weak gels or structurally compromised networks with reduced nutrient retention capacity. This study establishes the regulation of shear rate to control Mg^2+^ release rate as an effective strategy for producing premium selenium-enriched tofu with synchronized optimization of texture and nutritional value, providing new insights for improving the overall quality of functional plant-based protein gels.

## 1. Introduction

Selenium (Se) is an essential trace element for humans, with crucial physiological functions such as participation in glutathione peroxidase synthesis, regulation of antioxidant stress, and immune modulation having been extensively documented [1,2]. Due to widespread dietary selenium inadequacy, particularly in regions with low soil selenium levels or limited selenium-rich food consumption, food-based strategies that deliver selenium in a stable, bioavailable form have gained increasing attention [3]. Among these, selenium-enriched tofu has shown potential as an effective delivery system due to its ability to incorporate selenium in a stable, bioavailable form. This is because soybean proteins interact non-covalently with organic selenium species, such as selenomethionine, potentially enhancing selenium bioavailability [4,5]. Conventional brine-induced tofu coagulation relies on magnesium ions (Mg^2+^) to form soy protein gel networks. Mg^2+^ facilitates protein aggregation by reducing electrostatic repulsion and forming ionic bridges with negatively charged groups on the protein surface, promoting network formation [6]. However, the rapid action of Mg^2+^ in traditional brine systems leads to non-cooperative, kinetically dominated protein aggregation, limiting conformational adjustments and structural rearrangement [7,8]. Consequently, gel networks with loose, heterogeneous microstructures and enlarged pore sizes are often formed, with fewer effective protein binding sites and increased diffusion pathways, leading to organic selenium migration and loss during processing, which reduces selenium retention [9,10].

Substantial progress has been made in tofu quality enhancement through the development of diversified coagulation systems, each employing distinct mechanisms for targeted quality improvement. In enzyme-assisted coagulation, synergistic designs are increasingly employed. For instance, transglutaminase (TG), in combination with magnesium chloride (MgCl_2_) or calcium sulfate (CaSO_4_), follows a sequential strategy of enzymatic pre-crosslinking followed by salt-triggered coagulation, which promotes higher yield, improved water-holding capacity, and potentially better protein digestibility [11,12]. For organic-acid-induced coagulation, research has primarily focused on rational co-formulation. The co-application of glucono-δ-lactone (GDL) with hydrophilic polysaccharides strengthens gel hardness, elasticity, and improves water retention by fine-tuning the acidification profile and reinforcing network architecture [13]. In salt-induced coagulation, attention has shifted from coagulant selection to controlling coagulation kinetics. Systematic modulation of Ca^2+^/Mg^2+^ concentration and temperature allows for directed aggregation and network evolution, optimizing gel microstructure and macroscopic texture [7]. A particularly innovative strategy involves the use of emulsion-based coagulants for functional tofu production, leveraging their unique interfacial stability and encapsulation-controlled release capabilities [14]. As demonstrated in the relevant research, these systems effectively modulate protein aggregation kinetics through delayed ion release, significantly improving textural uniformity while refining gel network porosity [9,15,16]. More recent advancements in emulsion structural engineering, such as W/O/W high-internal-phase emulsions (HIPEs) incorporating soy protein isolate (SPI) in the inner aqueous phase, have been shown to increase yield and produce a more homogeneous gel network with lower hardness [16]. As demonstrated in our previous work, the optimization of magnesium-ion emulsion systems significantly improves water retention and sensory properties by fostering dense gel network formation via controlled ion release [17]. Our latest findings further indicated that the synergistic application of ultrasound pretreatment with emulsion coagulants provides enhanced optimisation of protein gel properties and improved texture homogeneity [18]. Despite the establishment of numerous technological approaches for enhancing tofu gel characteristics and textural properties, the synergistic relationship between process regulation and maximising nutrient retention in functional tofu (e.g., selenium-enriched tofu) remains under-explored. Specifically, the role of magnesium ion (Mg^2+^) release kinetics (particularly release rate) in mediating protein gelation behaviour and the retention of nutrients such as selenium has not been systematically elucidated; the causal relationship between release kinetics and final product quality remains unclear, directly resulting in consistently low retention rates of key nutrients like organic selenium.

In order to overcome this bottleneck, the present study developed a magnesium-loaded water-in-oil (W/O) emulsion coagulation system. This system has been designed to modulate the release kinetics of Mg^2+^ by regulating the shear rate. The central hypothesis of this study is that a moderate, shear-rate-controlled release of Mg^2+^ promotes the formation of an ordered soybean protein gel network, thereby enhancing nutrient retention. To test this hypothesis, the objectives are to characterize the release kinetics of Mg^2+^ as a function of shear rate, elucidate the mechanisms by which this release orchestrates protein aggregation and gelation, and assess its systematic impact on the retention of selenium and other nutrients. This study provides theoretical support and actionable solutions for the industrial development of high-quality, selenium-enriched tofu.

## 2. Materials and Methods

### 2.1. Materials

Selenium-enriched soybeans (protein content: 30.87%, Se content: 398.32 µg/kg) were provided by Hubei Tujia’ai Food Development Co., Ltd. (Yichang, China). The commercial sunflower oil employed in the study was obtained from Jiage Investment (China) Co., Ltd. (Zhengzhou, China). All chemicals, including MgCl_2_·6H_2_O, 5-5′-dithiobis-(2-nitrobenzoic acid) (DTNB), and polyglycerol poly-ricinoleate (PGPR), were obtained from Shanghai Macklin Biochemical Co., Ltd., (Shanghai, China) with the exception of petroleum ether, which was obtained from Tianjin Comio Chemical Reagent Co., Ltd. (Tianjin, China).

### 2.2. Preparation of Emulsion Coagulant

A water-in-oil (W/O) emulsion was prepared by mixing an aqueous phase containing 1.5 mol/L MgCl_2_ with sunflower oil containing 1.3% polyglycerol polyricinoleate (PGPR) in a 40:60 (water phase to oil phase) ratio. The mixture was stirred for 20 min to ensure uniformity. The primary emulsion was then subjected to high-speed shearing at 11,000 r/min for 3 min, followed by sonication at 176 W for 5 min. The resulting emulsion was stored at 4 °C for subsequent use [17].

### 2.3. Determination of the Release Kinetics of Emulsion Coagulants

The release kinetics of Mg^2+^ were monitored in real time using conductivity measurements. First, the conductivity of MgCl_2_ solutions at different concentrations was measured using a conductivity meter, and a standard curve relating concentration (y, g/L) to conductivity (x, mS/cm) was established by the following equation:

(1) $$y=0.0261x^{2}+0.4089x-0.0124\;(R^{2}=0.9992)$$

To simulate the tofu coagulation process, the prepared W/O emulsion was dispersed in distilled water and subjected to high-shear processing at shear rates ranging from 5000 to 14,000 r/min (in intervals of 1000 r/min) to trigger Mg^2+^ release. During the release process, the conductivity of the system was recorded every 15 s, and the conductivity values were converted into MgCl_2_ concentration using the previously established standard curve, allowing the real-time Mg^2+^ release profile to be obtained. For clarity, emulsion samples at different shear rates were labeled as R-n, where n represents the shear rate in units of 10^3^ r/min (e.g., R-10 corresponds to a shear rate of 10,000 r/min). This study aimed to characterize the temporal availability of Mg^2+^ during the coagulation process via kinetic analysis, rather than measuring the final total magnesium content in the tofu. Each sample was measured in triplicate to ensure data reliability [17].

### 2.4. Preparation of Soymilk

The cleaned soybeans were mixed with deionized water at a mass ratio of 1:3. Following this, the mixture was soaked at 25 °C for 12 h. Thereafter, the soaked beans were subjected to a grinding process with seven times their amount of deionized water. This process was carried out employing a high-speed blender (DJ10X-D285, Joyoung Co., Ltd., Hangzhou, China) for a duration of 2 min. The resulting slurry was filtered through a 100-mesh sieve to obtain raw soymilk. The raw soymilk was subsequently subjected to heating to boiling point and maintained at this temperature for a period of 5 min, thereby yielding cooked soymilk [17].

### 2.5. Determination of Protein Precipitation Kinetics

The cooked soymilk was subsequently permitted to cool to 80 °C. A coagulant was added to the mixture, which was then subjected to high-shear mixing at rates ranging from 6000 to 12,000 r/min (at 1000 r/min intervals) for a period of 20 s. The resulting samples were designated W/O-6, W/O-8, and so forth, with the numeral denoting the applied shear rate in thousands of r/min (for example, W/O-10 corresponds to a shear rate of 10,000 r/min). Subsequent to the process of shearing, the mixtures were permitted to stand at ambient temperature for periods ranging from one to 20 min. Subsequently, the samples were subjected to a centrifugal process, after which the soluble protein concentration in the resulting upper layer was measured [17]. The protein precipitation amount was calculated using the following equation:

(2) $$Protein\;precipitation\;amount\;(\%)=\frac{C_{0}-C_{t}}{C_{0}}\times 100$$

In this experiment, the soluble protein concentration in the supernatant at a given time (*C_t_*_)_ is measured and compared with the initial soluble protein concentration in soymilk (*C*_0_).

### 2.6. Preparation of Selenium-Enriched Tofu

The cooked soymilk (Section 2.1) was coagulated and molded at 80 °C. Specifically for the experimental groups, 9 mL of W/O emulsion coagulant was slowly added to the soymilk, followed by stirring at 6000, 8000, 10,000, or 12,000 r/min for 20 s to induce coagulation via Mg^2+^ release at different rates. For the control group, 15 mL of MgCl_2_ solution (0.6 mol/L) was added, and the mixture was stirred for 30 s to complete coagulation. All samples were then maintained at 80 °C for 20 min to allow gelation. The resulting curd was transferred to a cloth-lined mold and pressed at 28 g/cm^2^ for 60 min. The tofu prepared with MgCl_2_ solution was designated as MgCl_2_, whereas tofu prepared with the W/O emulsion coagulant was labeled according to stirring speed as W/O-6, W/O-8, W/O-10, and W/O-12 [17].

### 2.7. Sample Preparation for Analysis

Fresh tofu samples were cut into small pieces and freeze-dried under vacuum to constant weight. The dried samples were then ground into a fine powder and passed through a 60-mesh sieve to ensure sample homogeneity. The resulting freeze-dried tofu powder was stored in a desiccator until use and was used for subsequent sulfhydryl content determination, secondary structure analysis, and nutrient analyses, including protein, fat, selenium, total flavonoid, saponin, and total phenolic content.

### 2.8. Determination of Free Sulfhydryl Content (SH_F_)

The free sulfhydryl (SH) content was determined by the following procedure. Initially, samples were dissolved in distilled water (10 mg/mL). Subsequently, the samples were amalgamated with a Tris-Gly buffer and DTNB reagent (4 mg/mL). Following this, the samples were measured at 412 nm on a UH5300 spectrophotometer (Hitachi, Tokyo, Japan). A solution devoid of DTNB was used as the blank [19]. The SH content (μmol/g) was calculated using the following equation:

(3) $$SH_{F}(\mu mol/g)=\frac{A_{412}\times 10^{9}}{\varepsilon \times l\times C}$$

In this equation: *A*_412_ represents the absorbance at 412 nm, with no units, measured using a spectrophotometer; *ε* is the molar absorptivity (extinction coefficient) at 412 nm, with units of M^−1^·cm^−1^, and is equal to 13,600 M^−1^·cm^−1^; *l* is the path length through the sample, typically 1 cm; *C* is the protein concentration in the sample, expressed in mg/mL.

### 2.9. Fourier Transform Infrared Spectroscopy (FTIR)

Freeze-dried tofu samples were mixed with KBr at a 1:40 ratio and pressed into pellets for FTIR analysis. Spectra were recorded in the range of 4000–400 cm^−1^ with a resolution of 4 cm^−1^ and 32 scans per sample. The FTIR analysis focused on the amide I region (1600–1700 cm^−1^), which is sensitive to protein secondary structures [4]. The data were processed using PeakFit V4.12 software, with baseline correction and normalization to the amide I band, allowing for the quantification of secondary structure components, such as α-helix, β-sheet, β-turn, and random coil structures.

### 2.10. Texture Profile Analysis (TPA)

Tofu samples were analyzed using a texture analyzer (TA.XT Plus, Stable Micro Systems, Godalming, UK) in TPA mode with Exponent software (Version 6.1.26.0, Stable Micro Systems, Godalming, UK). Cubic samples were compressed to 50% of their original height using a cylindrical probe (P/36R) with a 5 g trigger force. Compression speeds were 2 mm/s before and after compression, with a 1 mm/s test speed and a 5-s interval between cycles. All tests were conducted at room temperature (25 °C), with triplicate measurements for reproducibility. Key TPA parameters, including hardness, springiness, cohesiveness, gumminess, and chewiness, were automatically calculated by the software [20].

### 2.11. Determination of Water Content

Moisture content was determined following a modified procedure from previous studies [21]. Approximately 2 g of fresh tofu sample (weighed to ±0.0001 g) was placed in a pre-dried aluminium dish and dried in a forced-air oven at 105 °C for 3 h. The drying process was repeated until constant weight was achieved, with weight measurements taken at 30-min intervals. The sample was cooled in a desiccator before each weighing.

### 2.12. Scanning Electron Microscopy (SEM)

The microstructure of tofu gel was examined using SEM (Thermo Scientific, Wlatham, MA, USA). Samples measuring 1 cm × 1 cm × 1 cm were fixed in 2.5% glutaraldehyde for 24 h. Thereafter, they were dehydrated through an ethanol gradient (60–100%), freeze-dried, and then cross-sections were observed [17].

### 2.13. Determination of Protein Content in Selenium-Enriched Tofu

Protein content was determined according to AOAC Official Method 991.20 using the Kjeldahl method. Approximately 0.50 g of dried tofu powder (accurate to 0.1 mg) was weighed and digested with concentrated sulfuric acid (H_2_SO_4_) in the presence of potassium sulfate (K_2_SO_4_) and a selenium catalyst. After the digest became clear, digestion was continued for an additional 30 min. The digest was then cooled, alkalized, and distilled; the liberated ammonia was captured in a boric acid solution containing a mixed indicator and subsequently titrated with a standardized hydrochloric acid (HCl) solution. A reagent blank was processed in parallel to correct the results. Nitrogen content (N%) was calculated from the difference in acid consumption between the sample and blank, the titrant concentration, and the sample mass, and protein content was obtained by multiplying N% by a conversion factor of 6.25. All measurements were performed in triplicate.

### 2.14. Determination of Fat Content in Selenium-Enriched Tofu

Fat content was determined by Soxhlet extraction, following the principles of AOAC Official Method 920.39 (Fat (Crude) or Ether Extract in Animal Feed) with modifications for soybean products. Clean receiving flasks were dried at 100 ± 1 °C to constant weight (difference between two consecutive weighings ≤ 0.5 mg), cooled in a desiccator, and weighed. Approximately 2.00 g of dried tofu powder (accurate to 0.1 mg) was placed in an extraction thimble and extracted with petroleum ether (boiling range 30–60 °C) for 4 h at a siphoning rate of at least four cycles per hour. After extraction, the solvent was evaporated, and the receiving flask was dried at 80 ± 1 °C for 1 h, cooled in a desiccator, and reweighed. The drying–cooling–weighing cycle was repeated until constant weight was achieved (≤0.5 mg). Fat content was calculated from the mass difference of the receiving flask before and after extraction relative to the initial sample mass and reported on a dry-weight basis. All samples were analyzed in duplicate, with the absolute difference between the two determinations required to be ≤1% of their mean value.

### 2.15. Determination of Selenium Content in Selenium-Enriched Tofu

Selenium content was quantified in tofu powder using inductively coupled plasma mass spectrometry (ICP-MS), following the procedure described in ISO 17294-2:2023 [22]. Approximately 5 g of freeze-dried tofu powder was accurately weighed and digested with nitric acid using a microwave digestion system. The digestion program followed a gradient temperature increase of 120 °C, 150 °C, and 190 °C, each held for 5 min. After digestion, the sample was transferred to a 50 mL volumetric flask and diluted with ultrapure water. Selenium was measured by ICP-MS with the following instrumental parameters: radio frequency power of 1500 W, carrier gas (argon) flow rate of 0.8 L/min, and auxiliary gas flow rate of 0.4 L/min. An appropriate internal standard was used to correct for matrix effects and signal drift. Each sample was analyzed in triplicate to ensure accuracy.

### 2.16. Determination of Total Flavonoid Content in Selenium-Enriched Tofu

Total flavonoid content was determined using the aluminum chloride colorimetric method [23]. For sample pretreatment, 0.5 g of tofu powder was extracted with 5 mL of absolute ethanol under dark conditions using ultrasonic treatment for 1 h, followed by centrifugation at 5000 r/min for 20 min. The supernatant was collected, and the residue was extracted once more under identical conditions. The combined extracts were diluted to 25 mL and stored at 4 °C prior to analysis.

Rutin was used as the reference compound for constructing the calibration curve. An accurately weighed 5 mg portion of rutin standard was dissolved in absolute ethanol and diluted to 10 mL. Aliquots of 0.25, 0.5, 1.0, 1.5, 2.0, and 2.5 mL of the standard solution were transferred into separate 10 mL test tubes. Subsequently, 0.5 mL of 5% sodium nitrite solution was added to each tube, mixed thoroughly, and allowed to react for 10 min. This was followed by the addition of 0.5 mL of 10% aluminum nitrate solution, mixing, and a further incubation for 10 min. Finally, 5 mL of 5% sodium hydroxide solution was added, and the mixtures were mixed well and allowed to react in the dark for 15 min. Absorbance was measured at 510 nm using a spectrophotometer, with the reagent blank used for zero adjustment. Sample extracts were analyzed using the same procedure, and the results were expressed as mg/g. The rutin calibration curve was established according to the following equation:

(4) $$y=0.2775x-0.009\;(R^{2}=0.9999)$$

where *y* is the absorbance and *x* is the rutin content, expressed in mg/g.

### 2.17. Determination of Saponin Content in Selenium-Enriched Tofu

Saponin content was determined using the vanillin–ethanol–sulfuric acid colorimetric method, with oleanolic acid as the standard [24]. The sample pretreatment followed the procedure described in Section 2.16, and the calibration curve was constructed using oleanolic acid as the reference standard. An accurately weighed 10 mg portion of oleanolic acid was dissolved in ethanol and diluted to 50 mL in a volumetric flask. Aliquots of 0.1, 0.2, 0.3, 0.4, 0.5, and 0.6 mL of the standard solution were transferred into separate 10 mL stoppered test tubes and evaporated to dryness in a 60 °C water bath. Subsequently, 0.5 mL of 10% vanillin–ethanol solution and 5 mL of sulfuric acid were added to each tube. After thorough mixing, the reaction mixtures were incubated in a 60 °C water bath for 1 h and then immediately cooled in an ice bath. A parallel sample without the addition of oleanolic acid was used as the blank. Absorbance was measured at 450 nm, and the calibration curve was constructed accordingly. Sample extracts were analyzed following the same procedure, and the results were expressed as mg/g. Each sample was measured in triplicate to ensure data reliability The oleanolic acid calibration curve was described by the following equation:

(5) $$y=0.0026x-0.009\;(R^{2}=0.9926)$$

where *y* is the absorbance and *x* is the oleanolic acid content, expressed in mg/g.

### 2.18. Determination of Total Phenol Content in Selenium-Enriched Tofu

Total phenol content was determined using the Folin–Ciocalteu method, as described in ISO 14502-1:2005/Cor 1:2006 [25]. The sample pretreatment followed the procedure described in Section 2.16, and the calibration curve was constructed using gallic acid as the reference standard. Briefly, 0.1 g of gallic acid was dissolved in 10 mL of absolute ethanol and diluted to 100 mL with distilled water. Aliquots of 1, 2, 3, 4, 5, and 6 mL of the standard solution were each diluted to 10 mL to obtain gallic acid solutions at different concentrations. For the colorimetric reaction, 1 mL of each standard solution was mixed with 1 mL of Folin–Ciocalteu reagent and allowed to stand for 5 min, followed by the addition of 5 mL of 7.5% sodium carbonate solution. The reaction mixture was incubated at 50 °C, and absorbance was measured at 765 nm against a reagent blank. Each sample was measured in triplicate to ensure data reliability The gallic acid calibration curve was described by the following equation:

(6) $$y=0.1774x+0.0503\;(R^{2}=0.9959)$$

where *y* is the absorbance, *x* is the concentration of gallic acid (mg/mL).

### 2.19. Statistical Analysis

Statistical analysis was conducted using IBM SPSS Statistics 26. Prior to analysis, the normality of the data was assessed with the Shapiro–Wilk test, and the homogeneity of variances was tested using Levene’s test. One-way analysis of variance (ANOVA), followed by Duncan’s post hoc test (*p* < 0.05), was used to perform multiple comparisons. All data are presented as the mean ± standard deviation (SD). Figures were generated using Origin 2021.

## 3. Results and Analysis

### 3.1. Effects of Shear Rate on the Kinetics of Coagulant Release and Protein Aggregation in Emulsion Systems

#### 3.1.1. Mg^2+^ Release from Emulsion Droplets Under Different Shear Rates

**Figure 1:**
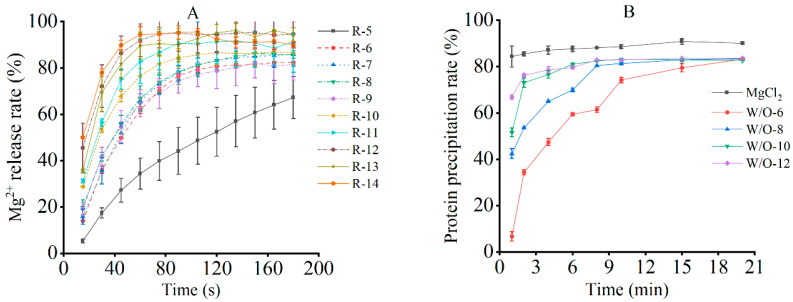
Effects of shear rate on the release of emulsion coagulant (**A**) and protein coagulation (**B**). The results are expressed as the mean value ± standard deviation.

In emulsion systems, the application of shear forces disrupts interfacial films, inducing droplet deformation and coalescence. This, in turn, has been demonstrated to significantly affect microstructural stability and promote Mg^2+^ release [26]. As demonstrated in Figure 1A, the release of Mg^2+^ increased with both the shear rate and duration, owing to cumulative structural damage. However, except for 5000 r/min, the release profiles exhibited three distinctive phases: an initial rapid rise (film rupture), a subsequent deceleration (limited coalescence), and eventual saturation (structural breakdown). It was demonstrated that higher shear rates accelerated both the initial release and saturation time. This is consistent with the mechanism of rapid emulsion disintegration under intense shear, as reported by Anvari and Joyner [27].

At 5000 r/min, Mg^2+^ release increased continuously without reaching saturation (Figure 1A), suggesting that the applied shear force was insufficient to fully disrupt droplet interactions [28]. Furthermore, the observed maximum release level of approximately 90% can be attributed to two factors. First, the formation of a stable oil-water interfacial film by excess PGPR (polyglycerol polyricinoleate) has been shown to hinder the instantaneous release of aqueous microspheres during disruption. Second, the potential re-encapsulation of released aqueous microspheres by the oil phase has been demonstrated to limit Mg^2+^ dispersion [29,30]. Consequently, shear rates exceeding 5000 r/min are required to ensure sufficient Mg^2+^ availability for effective tofu coagulation. As demonstrated in Figure 1A, although the ultimate extent of Mg^2+^ release remained comparable across shear rates ranging from 6000 to 12,000 r/min, the release kinetics, including the initial release rate and the time required to reach saturation, varied substantially. Notably, by the end of the 20-s shear treatment, corresponding to the early stage of Mg^2+^ release, distinct differences among shear rates were already evident from the release profiles (Figure 1A). This indicates that the applied shear duration was sufficient to establish differential Mg^2+^ availability prior to subsequent heat-induced coagulation. Although Mg^2+^ release continued after shear termination, the subsequent release kinetics tended to converge, indicating that shear rate primarily modulates the temporal availability of Mg^2+^ during coagulation rather than the ultimate extent of Mg^2+^ release. On this basis, shear rates of 6000, 8000, 10,000, and 12,000 r/min were selected for subsequent experiments to elucidate how differences in release kinetics influence protein gelation behavior and final tofu quality.

#### 3.1.2. Soy Protein Aggregation Following Mg^2+^ Release

The precipitation of soy protein has been shown to be highly dependent on the Mg^2+^ concentration. During the process of brine coagulation, the application of heat in conjunction with metal ions has been demonstrated to induce protein pre-aggregation, resulting in the formation of primary aggregates that subsequently flocculate and ultimately sediment through intermolecular interactions [9,31,32,33]. This dynamic process is critical in determining the final texture and gel structure of tofu. The present study sought to evaluate the coagulation behaviour of the sample under investigation in the context of varying shear rates. To this end, the protein precipitation over time was measured. When MgCl_2_ was used as the sole coagulant, elevated levels of protein precipitation were attained in the early stages (Figure 1B), signifying expeditious Mg^2+^-induced crosslinking. Conversely, an emulsion-based coagulant system exhibited delayed precipitation, with initial levels being lower but gradually increasing before reaching a state of stability (Figure 1B). This finding was consistent with the Mg^2+^ release kinetics, thereby confirming the hypothesis that the rate of ion release is a regulator of protein aggregation.

The sustained-release behaviour of the emulsion is attributable to its physicochemical properties. At lower shear rates, weaker shear forces preserve the structural integrity of the emulsion droplets; simultaneously, the system’s inherently higher viscosity and resulting restricted flowability synergistically slow the outward migration and diffusion of Mg^2+^, creating a moderate concentration gradient [32]. Within this controlled release environment, Mg^2+^ facilitates the process of ordered protein aggregation through a combination of bridging and charge screening, thereby contributing to the formation of stable gels. However, at speeds exceeding 8000 r/min, intense shear stress induces deformation and rupture of the W/O emulsion droplets, causing the rapid release of encapsulated Mg^2+^. Simultaneously, the inherent shear-thinning behavior of the emulsion reduces system viscosity, further accelerating ion diffusion [34]. This combined effect results in a sharp local concentration imbalance. This phenomenon is known as “disordered aggregation”, which occurs when magnesium (Mg^2+^) saturation leads to the formation of over-crosslinked yet weak dense zones. This, in turn, results in flocculation and a subsequent decline in texture [18,34,35].

### 3.2. Effects of Emulsion Coagulant on Free Sulfhydryl Content in Selenium-Enriched Tofu

**Figure 2:**
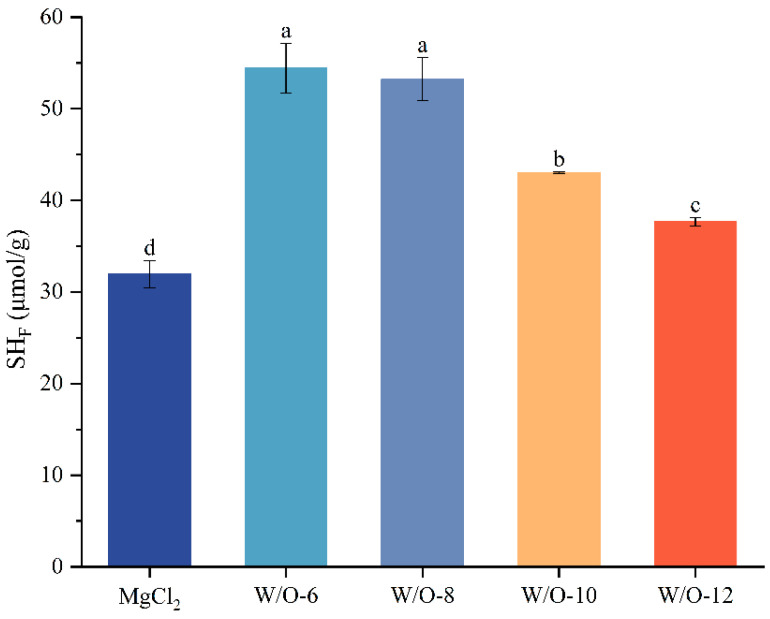
Effects of shear rate on free sulfhydryl content in selenium-enriched tofu. The results are expressed as the mean value ± standard deviation. Mean values followed by different letters are significantly different at *p* < 0.05.

The dynamic changes of sulfhydryl groups (-SH) in protein molecules directly regulate protein aggregation and gel network formation. The present study demonstrates a strong correlation between Mg^2+^ release kinetics and sulfhydryl group utilisation. The findings demonstrated that the free sulfhydryl content in selenium-enriched tofu prepared with the traditional coagulant (MgCl_2_ solution) was 31.94 μmol/g. In comparison, W/O-6 and W/O-8 emulsions formulated at low shear rates substantially elevated the free sulfhydryl content in selenium-enriched tofu (by 22.48 and 21.30 μmol/g, respectively, i.e., reaching 54.42 and 53.24 μmol/g, Figure 2). This increase can be attributed to the sustained-release properties of these emulsions, which prolong protein crosslinking and enhance sulfhydryl exposure. These exposed free sulfhydryls act as active crosslinking sites, enhancing gel network order and mechanical strength [36]. Conversely, the rapid release of Mg^2+^ from W/O-10 and W/O-12 emulsions resulted in the immediate formation of protein over-aggregates (Figure 1B), leading to the burial of sulfhydryls through steric hindrance or the oxidation of sulfhydryls into disulfide bonds. This process resulted in a substantial decrease in free sulfhydryl levels to 43.02 and 37.64 μmol/g (Figure 2). This outcome is consistent with earlier research that has reported a decrease in the free sulfhydryl content of soy protein to 35.2–39.7 μmol/g due to excessive crosslinking [12,17,36]. The present findings demonstrate that the rate of Mg^2+^ release exerts a critical influence on protein crosslinking: moderate, sustained release optimises sulfhydryl utilisation, while excessive speed compromises crosslinking sites.

### 3.3. Effects of Emulsion Coagulant on the Secondary Structure of Selenium-Enriched Tofu

**Figure 3:**
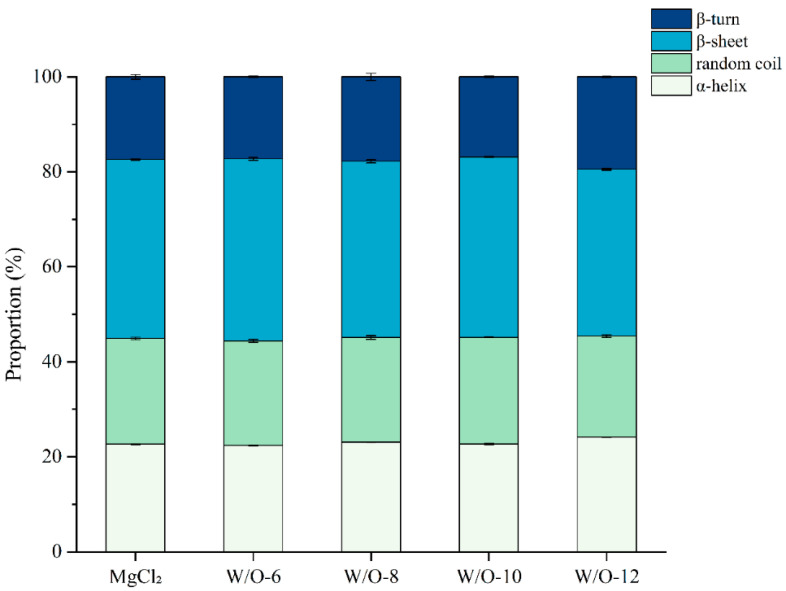
Effects of emulsion coagulant on the secondary structure content in selenium-enriched tofu. The results are expressed as the mean value ± standard deviation.

It has been demonstrated that dynamic changes in β-sheet content can exert a significant influence on gel network quality in soy protein gels [37]. The FTIR spectra were baseline-corrected and normalized to the amide I band (1600–1700 cm^−1^). The relative proportions of α-helix, β-sheet, β-turn, and random coil structures were determined by integrating the corresponding peak areas. This approach ensured the accurate quantification of secondary structure components and allowed for a clear comparison across different processing conditions. This analysis demonstrated that the W/O-6 system prepared at 6000 r/min increased the β-sheet content to 38.34%, which was significantly higher than the control group (37.68%, Figure 3). This phenomenon is closely related to the dual mechanism of slow Mg^2+^ release (Figure 1A). It has been demonstrated that this process prolongs the time taken for orderly protein aggregation, while maintaining the system’s maximum free sulfhydryl content (Figure 2). This provides sufficient space for conformational adjustments and reactive sites for intermolecular crosslinking. These findings are consistent with the controlled-release theory, which posits that moderate Mg^2+^ release facilitates orderly protein assembly. They also provide molecular-level confirmation of recent research conclusions that dynamic changes in β-sheet content significantly impact the quality of soy protein gel networks [38].

It is noteworthy that the proportion of β-sheets decreased in a stepwise manner with rising shear rates (Figure 3). This phenomenon is closely linked to the rapid release of Mg^2+^ ions under high-shear conditions. Specifically, the sudden increase in Mg^2+^ concentration has been shown to induce non-specific protein crosslinking, thereby disrupting the hydrogen bonds required for β-sheet formation (Figure 1A and Figure 2). Concurrently, the rapid entanglement of proteins leads to conformational disorder, while the sharp decline in free sulfhydryl content creates a critical shortage of cross-linking sites, which hinders the development of ordered β-sheet structures collectively [17].

### 3.4. Effects of Emulsion Coagulants on the Textural Properties of Selenium-Enriched Tofu

The study demonstrates considerable variations in the textural characteristics of selenium-enriched tofu, contingent on Mg^2+^ release kinetics (Table 1). Conventional MgCl_2_ coagulation yielded tofu with the highest hardness (321.30 g), however, it demonstrated textural deficiencies, including excessive chewiness (228.96) and inadequate resilience (38.73%), which is a consequence of accelerated gelation. In contrast, the W/O-8 emulsion system was found to optimise Mg^2+^ release, thereby facilitating the formation of a “magnesium bridge” with soy protein carboxyl groups. This enhanced crosslinking density has been demonstrated to promote dynamic sulfhydryl reorganization and β-sheet formation, resulting in a homogeneous three-dimensional network. Consequently, the tofu exhibited reduced hardness and chewiness (*p* > 0.05), accompanied by a significant increase in resilience (*p* < 0.05), in comparison to conventional methodologies [17]. Furthermore, the extent of the modification of both resilience and springiness is greater than that achieved using typical acid-based coagulants (e.g., GDL) or enzyme-based coagulants (e.g., transglutaminase), as documented in the literature [21,39]. It is noteworthy that tofu prepared using the W/O-6 system exhibited significantly lower hardness (126.91 g), representing a 60.5% reduction compared with the MgCl_2_ control. This behavior can be attributed to insufficient protein crosslinking associated with delayed Mg^2+^ release. Although such release kinetics are favorable for establishing a mild concentration gradient, the limited ion flux may have resulted in a reduced crosslinking density, leading to a loose and porous gel structure with elevated water retention. Consequently, the tofu exhibited a soft and collapse-prone texture [36]. This finding is consistent with our previous study, which demonstrated that insufficient ion release leads to decreased gel strength, further supporting the critical role of the Mg^2+^ release rate in determining tofu texture [18].

### 3.5. Effects of Emulsion Coagulants on the Microstructure of Selenium-Enriched Tofu

**Figure 4:**
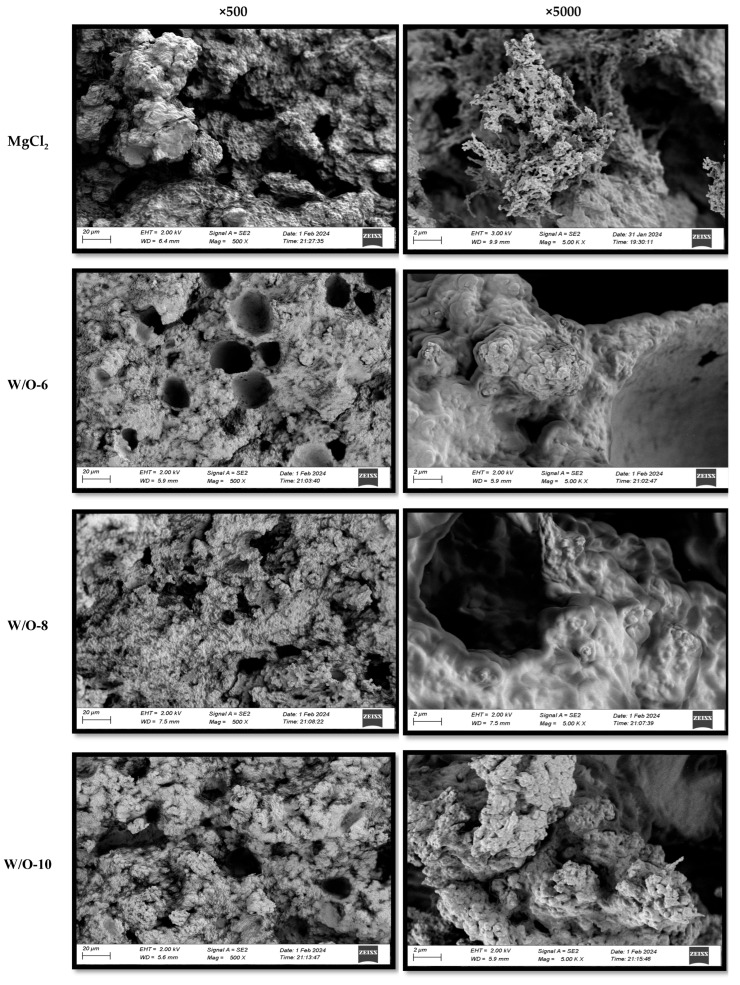

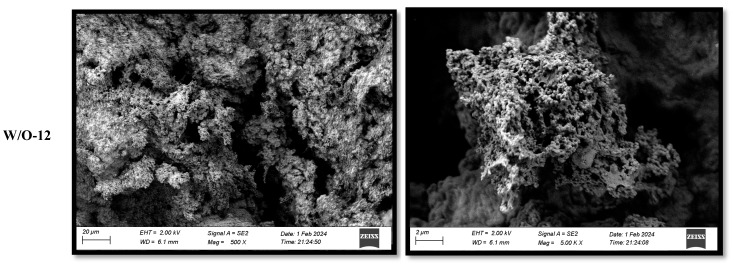
Effects of emulsion coagulant on the microstructure of selenium-enriched tofu.

SEM analysis demonstrated that emulsion coagulants caused substantial modification to the microstructure of selenium-enriched tofu. As demonstrated in Figure 4, traditional MgCl_2_-coagulated tofu exhibited irregular honeycomb structures. This phenomenon can be attributed to the rapid release of Mg^2+^ ions, which induced protein crosslinking. This, in turn, resulted in the formation of large pores and heterogeneous networks [17]. Conversely, the W/O-8 system (emulsion coagulant) exhibited optimal structural properties, characterised by sustained Mg^2+^ release, which prolonged protein crosslinking, resulting in the formation of a dense gel network with small, uniform pores and smooth surfaces (Figure 4). The W/O-6 system produced structures that were less dense but still superior to conventional methods [15]. The refined porous structure was found to significantly enhance hydration properties and is expected to improve nutrient bioavailability by effectively entrapping and stabilizing bioactive compounds within the gel network [15,32,40].

The accelerated Mg^2+^ release resulted in an increase in protein aggregate size, a reduction in pore number, and an enlargement of the pore diameter (Figure 4). This phenomenon was particularly evident at elevated shear rates (>8000 r/min), where a substantial enlargement of pores (Figure 4) was observed to correlate with a deterioration in textural properties (Table 1).

### 3.6. Impacts of Emulsion Coagulants on Water, Protein and Fat Retention in Selenium-Enriched Tofu

**Figure 5:**
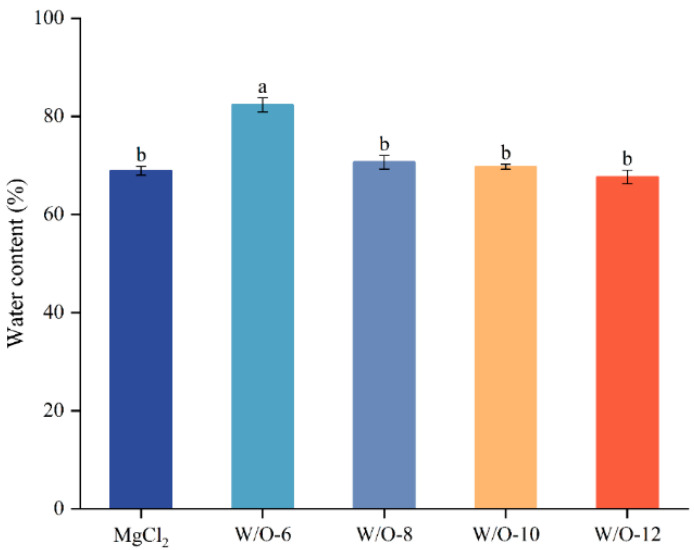
Effects of emulsion coagulant on the water content of selenium-enriched tofu. The results are expressed as the mean value ± standard deviation. Mean values followed by the different letters are significantly different at *p* < 0.05.

The water content of tofu plays a critical role in determining gel structure and overall quality, whereas protein and fat retention rates are key indicators of nutritional quality. Compared with conventional MgCl_2_ coagulation, tofu produced under W/O-6 and W/O-8 conditions exhibited higher water, protein, and fat retention (Table 2 and Figure 5). This comprehensive improvement is attributed to the differential release behavior of Mg^2+^ under varying shear conditions and the corresponding network formation mechanisms. Under W/O-6 (lower shear) conditions, the slow release of Mg^2+^ establishes a relatively mild coagulation environment, which helps preserve the native conformation and structural integrity of proteins, thereby reducing protein loss caused by rapid denaturation and aggregation [17,33]. The W/O-6 system produced structures that were less dense (i.e., more open and interconnected) than those of W/O-8, yet still superior to the coarse, heterogeneous networks formed by conventional methods (Figure 4). This highly open and continuous porous architecture effectively retains water through physical accommodation [15,16]. This structural environment also favors the uniform dispersion and embedding of fat droplets within the protein matrix, contributing to the enhanced water and fat retention observed [16]. Under W/O-8 (optimized shear) conditions, the gradual and consistent release of Mg^2+^ has been demonstrated to facilitate salt bridge crosslinking. This process functions synergistically with disulfide bonds, which are facilitated by free sulfhydryl groups, as well as with the orderly assembly of proteins directed by β-sheets. The combined action of these interactions results in the formation of a stable, homogeneous, and structurally dense protein gel network (Figure 4). The uniform and fine pore structure significantly enhances water retention by improving water entrapment and immobilization through capillary forces and surface adsorption [33]. Concurrently, protein fixation within the tofu matrix is maximized through the dual mechanisms of physical entrapment (spatial hindrance) and chemical binding (salt-bridge crosslinking), thereby effectively minimizing protein loss [35]. Furthermore, moderate shear conditions have been shown to preserve protein integrity, thereby preventing the loss of nutrients that is induced by mechanical damage. The enhancement in fat retention can be ascribed to the inherent configuration of oleosin, which functions by encapsulating fats in a manner that impedes oxidative degradation. The slow coagulation process extends the time available for protein-lipid interaction, thereby facilitating the embedding of fat droplets into the gel network. Furthermore, an optimised emulsification system enhances fat dispersion stability, reinforcing its immobilisation in the gel structure [41]. The collective impact of these elements is to guarantee elevated fat retention.

However, under W/O-10 and W/O-12 conditions, water, protein, and fat retention decreased markedly (Table 2 and Figure 5). This phenomenon is attributed to the rapid gelation process, which results in the formation of a loose, porous structure with reduced entrapment efficiency (Figure 4). It has been demonstrated that increases in shear rates result in elevated levels of viscosity, which can consequently lead to a reduction in component adhesion. In addition, it has been demonstrated that excessive shear has the capacity to disrupt emulsion stability, thereby promoting fat droplet aggregation and phase separation. This, in turn, can result in a further reduction in fat retention [42].

### 3.7. Impacts of Emulsion Coagulants on Retention Rates of Selenium, Saponins, Phenolics, and Flavonoids in Selenium-Enriched Tofu

The presence of functional components in tofu, such as selenium, saponins, phenolics, and flavonoids, renders it a significant nutritional indicator due to its health benefits, including antioxidant, anti-inflammatory, and lipid-regulating properties. The findings of research conducted to date indicate that emulsion coagulants enhance the retention of these compounds, whereas rapid Mg^2+^ release reduces retention, contingent on gel structure, moisture, and component characteristics (Table 2). The slow Mg^2+^ release has been shown to promote the formation of denser soy protein gel networks, leading to the immobilisation of selenoproteins and phenolics via covalent bonds, hydrogen bonds, and hydrophobic interactions [9,40]. This process has been demonstrated to enhance the water-holding capacity, thereby minimising the loss of flavonoids and saponins (Figure 5 and Table 2). This mild gelation also stabilises Mg^2+^-phenolic complexes, significantly improving bioactive retention [43].

Conversely, under conditions of high-release (W/O-10 and W/O-12), the process of accelerated gelation gives rise to the formation of loose, porous structures (Figure 4). This phenomenon has been demonstrated to result in a reduction in selenium and saponin retention, whilst concurrently promoting thermal degradation or leaching of heat-labile phenolics and flavonoids [17,44]. Consequently, it can be deduced that the precise regulation of Mg^2+^ release, with the aim of optimising gel microstructure, will maximise bioactive retention and nutritional quality.

### 3.8. Discussion

**Figure 6:**
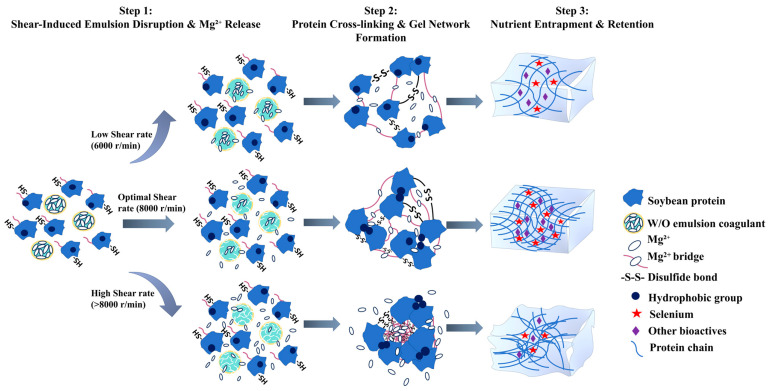
Mechanistic insight into shear-controlled Mg^2+^ release from W/O emulsion coagulant for synchronous optimization of gel properties and nutrient retention in selenium-enriched tofu.

The present study systematically investigated the mechanism by which shear rate influences the release kinetics of Mg^2+^ from W/O emulsions and the quality formation of tofu. The experimental findings have demonstrated that the shear rate functions as a pivotal processing parameter, thereby regulating the release behaviour of the emulsion coagulant. It is evident that by adjusting the shear rate, the Mg^2+^ release rate can be significantly modified, thereby affecting the protein gelation process (Figure 1A). In the context of optimal shear conditions, defined as a rate of 8000 revolutions per minute (r/min), the application of moderate shear forces resulted in a controlled disruption of the emulsion interface (Figure 6). This disruption facilitated a sustained and stable release of Mg^2+^ (Figure 1A and Figure 6). The process was found to be effective in synchronising with the conformational changes of proteins, thereby promoting their moderate unfolding and subsequent exposure of key active sites, including carboxyl groups, hydrophobic regions, and sulfhydryl groups. This dynamic coordination has been shown to facilitate an orderly protein assembly pathway: Initially, Mg^2+^ established a primary network framework through electrostatic interactions, which was subsequently consolidated by hydrophobic forces under suitable ionic conditions, and ultimately stabilized via disulfide bond formation (Figure 6).

Subsequent analysis, encompassing a range of shear conditions, further substantiated this mechanism. The weak gel strength observed at 6000 r/min was primarily due to excessively slow Mg^2+^ release, which prevented the formation of a sufficiently dense cross-linked network. Conversely, when the shear rate exceeded 8000 r/min, excessive shear forces caused rapid disruption of the emulsion interface, leading to substantial Mg^2+^ release (Figure 1A). This rapid ion release has been shown to disrupt the orderly process of protein assembly, inducing disordered aggregation and phase separation, which ultimately results in a coarse and porous microstructure (Figure 4). The research findings demonstrate that precise control of shear rate, as a pivotal processing parameter, facilitates the concurrent enhancement of tofu texture and nutritional quality, thus providing significant guidance for the development of novel functional soybean products.

## 4. Conclusions

This study systematically investigated the influence of shear rate (6000–12,000 r/min) on modulating magnesium ion (Mg^2+^) release from a W/O emulsion coagulant and its consequent effects on the quality of selenium-enriched tofu. The results establish shear rate as a critical parameter governing Mg^2+^ release kinetics, which in turn determines the microstructure, textural properties, and nutrient retention of the final product. At the optimal shear rate of 8000 r/min, a sustained release of Mg^2+^ facilitated a sequential and orderly protein assembly. This mechanism was characterized by a marked increase in free sulfhydryl group exposure and β-sheet formation, culminating in a uniform and dense three-dimensional network. This optimally engineered microstructure directly resulted in the resilience of selenium-enriched tofu increasing from 38.73% to 42.54%, and the selenium retention rate rising from 44.42% to 54.57%. Consequently, this approach successfully achieves the coordinated optimization of texture and nutritional quality. This study establishes a theoretical framework for advancing the development of selenium-enriched tofu.

## Figures and Tables

**Table 1:** TPA parameters of selenium-enriched tofu.

Samples	Hardness (g)	Chewiness	Cohesiveness	Springiness (%)	Resilience (%)
MgCl_2_	321.30 ± 32.90 ^a^	228.96 ± 34.24 ^a^	93.33 ± 3.14 ^bc^	0.76 ± 0.02 ^b^	38.73 ± 2.59 ^b^
W/O-6	126.91 ± 11.66 ^d^	99.63 ± 9.68 ^d^	98.09 ± 0.63 ^a^	0.80 ± 0.01 ^a^	43.91 ± 1.31 ^a^
W/O-8	298.67 ± 21.74 ^a^	222.91 ± 14.04 ^a^	95.88 ± 2.08 ^ab^	0.78 ± 0.01 ^b^	42.54 ± 0.61 ^a^
W/O-10	262.13 ± 27.07 ^b^	187.02 ± 23.02 ^b^	92.99 ± 0.88 ^c^	0.77 ± 0.01 ^b^	43.35 ± 0.05 ^a^
W/O-12	220.91 ± 33.23 ^c^	139.71 ± 23.83 ^c^	90.26 ± 1.33 ^d^	0.72 ± 0.02 ^c^	37.79 ± 1.21 ^b^

Results are expressed as mean value ± standard deviation. Mean values followed by different letters are significantly different at *p* < 0.05.

**Table 2:** Effects of emulsion coagulants on the nutrient retention in selenium-enriched tofu.

Samples	Proteins Retention Rate (%)	Fats Retention Rate (%)	Selenium Retention Rate (%)	Total Phenol Retention Rate (%)	Total Flavonoid Retention Rate (%)	Saponin Retention Rate (%)
MgCl_2_	57.86 ± 0.80 ^cd^	11.53 ± 0.29 ^b^	44.42 ± 1.01 ^c^	18.49 ± 1.03 ^c^	21.65 ± 0.66 ^e^	18.20 ± 0.10 ^e^
W/O-6	69.91 ± 0.36 ^b^	15.18 ± 0.32 ^a^	62.43 ± 0.37 ^a^	49.53 ± 4.86 ^a^	49.22 ± 2.14 ^a^	51.32 ± 0.22 ^a^
W/O-8	72.74 ± 0.52 ^a^	14.77 ± 0.16 ^a^	54.57 ± 2.93 ^b^	35.41 ± 3.93 ^b^	37.23 ± 1.07 ^b^	38.36 ± 0.27 ^b^
W/O-10	59.74 ± 0.10 ^c^	11.63 ± 0.01 ^b^	47.99 ± 1.66 ^c^	20.23 ± 2.47 ^c^	30.21 ± 0.52 ^c^	27.11 ± 0.18 ^c^
W/O-12	55.26 ± 2.03 ^d^	10.92 ± 0.12 ^c^	43.97 ± 2.20 ^c^	19.90 ± 1.50 ^c^	24.69 ± 0.81 ^d^	23.21 ± 0.01 ^d^

Results are expressed as mean value ± standard deviation. Mean values followed by different letters are significantly different at *p* < 0.05.

## Data Availability

The original contributions presented in this study are included in the article. Further inquiries can be directed to the corresponding author.
